# Radiomodifying action, Pharmacokinetic and Biodistribution of Ethyl 3, 4, 5-trihydroxybenzoate-Implication in development of radiomitigator

**DOI:** 10.1038/s41598-019-55316-2

**Published:** 2019-12-11

**Authors:** Pranav K Pandey, B. Ahmed, J. Prasad, M. Bala, H. A. Khan

**Affiliations:** 1 Division of Radiation Biology, Institute of Nuclear Medicine and Allied Sciences, Defence Research and Development Organisation, New Delhi, 110054 India; 2 Department of Pharmaceutical Chemistry, School of Pharmaceutical Education and Research, Jamia Hamdard, New Delhi, 110062 India; 3 Center for Translational and Clinical Research, Jamia Hamdard, New Delhi, 110062 India; 4 Formerly Scientist at Division of Radiation Biology, Institute of Nuclear Medicine and Allied Sciences, Defence Research and Development Organisation, New Delhi, 110054 India; 5 Department of Medical Elementology and Toxicology, School of Chemical and Life Sciences, Jamia Hamdard, New Delhi, 110062 India

**Keywords:** Enzyme mechanisms, Pharmacokinetics

## Abstract

Ethyl 3, 4, 5-trihydroxybenzoate (GAE) is a major bioactive constituent of *Hippophae Rhamnoides L*. leaves and extract prepared from *H. rhamnoides* leaves exhibited radioprotective and pharmacological activity. Radiomodifying properties of polyphenol compounds through free radical neutralizing have been reported earlier. However, to date pharmacokinetic (PK) and biodistribution of polyphenol compounds post ^60^Co-γ-irradiation (5 Gy) exposure have not been studied yet. The study aims to investigate the radio modifying and inflammatory action, PK and biodistribution of GAE at a radioprotective dose and changes, if any, induced after irradiation. Male C 57 BL/6 mice (28–30 g) were administered GAE (200 mg/kg b.wt) orally 15 minutes post to irradiation. Mice were sacrificed at 15, 30 min, 1,2,4,8 and 24 h. PK and biodistribution of GAE in plasma and tissues were studied. The radiomodifying potential was assessed in terms of mitigating NF-kB activity and SGOT, SGPT, urea and creatinine levels in liver and kidney post irradiation. Our study suggested the potential use of GAE as radiomodifying agent inhibits NF-kB expression and maintains the SGOT 24.10 ± 2.4, SGPT 36.01 ± 6.1 U/l, urea18.16 ± 0.003, and creatinine 1.05 ± 0.04 mg/dL upto 8 h in comparison to irradiated mice. Moreover, in biodistribution studies, showed that GAE crosses the blood-brain barrier and is found in brain tissue. Plasma level of GAE peaked at about 15 min, with C_max_ 4390.85 ± 285.20 in GAE and in 3391.78 ± 78.13 ng/mL in radiation + GAE-treated animals, Biodistribution resulted in the highest concentration to be found in liver and kidney. These radiomodifying and pharmacokinetic result may be useful for study of the bioactive mechanism associated with radiation injury and to develop a potent formulation of GAE for clinical application.

## Introduction

Ionizing radiation is a form of energy released from atoms and comprising various wavelength such as x-rays or gamma rays. The living organism is exposed to radiation sources on a daily basis either from natural or man-made sources. Globally more than 3600 million medical diagnostic radiology examinations was performed, more than 37 million nuclear medicine procedures were carried out and almost above 7.5 million radiotherapies for cancer patient were given^1^. Exposure of radiation either directly or indirectly penetrates living cell or tissue which induces oxidative stress due to overproduction of several free radicals and disturbed enzymatic activity at the cellular level. These free radicals reactive oxygen and nitrogen species (ROS and RNS) such as superoxide anion (O_2•_^-^), hydroxyl ion (OH^-^) and hydrogen ion (H^+^) attack nucleic acids, lipid membranes and proteins in the nucleus to produce secondary radicals and cause intracellular toxicity^2^. Radiation countermeasures have been divided into three classes radioprotector, radiomitigator and therapeutic. The countermeasure agents were administered in accordance to their classification, in radioprotector agent should be administered prior to exposure, in radiomitigator agent should be administered shortly after the exposure but before the onset of symptoms and in therapeutic agent should be administered after the injury and onset of physical symptoms^3,4^. Worldwide a great deal of time and money has been spent on the pre-clinical and clinical research with the aim of developing safe and efficacious radiomodifying agents; however, until this date none of them were found to be safe and nontoxic for human use^5,6^. After extensive screening of molecules and failure of most of these synthetic compounds, researchers turned their attention toward phytochemical and plant extracts to investigate radioprotective efficacy^7,8^. *Hippophae rhamnoides L*. (Seabuckthorn) *Elaegnaceae* is valuable plant has recently gained attention worldwide due to its nutritional and medicinal properties. Sea buckthorn (*Hippophae rhamnoides L*.) is nitrogen fixing deciduous shrub of cold arid region native to Europe and Asia^9^. *H. rhamnoides* leaves aqueous extract (SBL-1) showed radiation protection at a lethal dose of ionizing radiation^10^. Ethyl 3, 4, 5-trihydroxybenzoate (GAE) is commonly known as Gallic acid ethyl ester and one of the major bioactive constituent present in aqueous extract coded SBL-1^11^.

**Figure 1:**
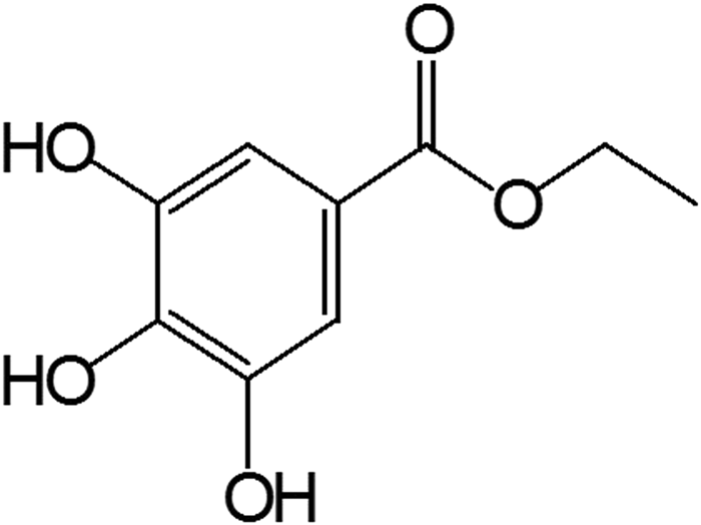
Gallic acid ethyl ester, C_9_H_10_O_5_ Molecular Weight: 198.17, m/z: 198.05 (100.0%), 199.06 (10.0%), 200.06 (1.5%) Elemental Analysis: C, 54.55; H, 5.09; O, 40.37.

GAE, C_9_H_10_O_5_ has molecular weight 198.17 g/mol (Fig. 1). It is a colorless or slightly yellow crystalline compound possessing anti-inflammatory^12^, antibacterial^13^, and anticancer properties^14^. It is a polyphenol compound derivative of the shikimic acid pathway. The secondary metabolites of plants, are a very good source for the discovery and development of lead molecule. GAE has been reported in extracts of some other plants also such as *Terminalia chebula*, which have shown neuroprotective effects^15^.

Changes in pharmacokinetic properties of polyphenols compounds induced by the exposure of the organism to ionizing radiation have been rarely studied. The present study aim is to investigate the radiomodifying action of GAE in pharmacokinetic and biodistribution studies and to observe changes in pharmacokinetics, and biodistribution of GAE due to whole body ^60^Co-γ radiation. The alteration in the level of GAE post irradiation is identified by observing changes in pharmacokinetic parameters and biodistribution patterns and by investigating liver and kidney function as well measurement of NF-kB activity in order to assess the inflammation associated with radiation exposure. The pharmacokinetic, biodistribution and radiomodifying study of GAE in animal model will help in understanding the GAE distribution mechanism as well further assist in achieving better application for the development of GAE as radiomodifying agents.

## Results

### Characterization of GAE isolated from Hippophae rhamnoides leaves

#### NMR Spectra

The ^1^H-NMR spectrum of GAE isolated from *Hippophae Rhamnoides* leaves exhibited and displayed a triplet three protons 3 H,t, (J = 6.8,7.2hz) and 4.19,1 H,d, (J = 6.8hz, CH_2_ a), 4.22, 1 H,d,(J = 7.2hz, CH_2_ b), were displayed. ^13^C NMR spectrum of GAE displayed a peak 14.7 due to methyl group (CH_3_), 60.4 due to CH_2_ group other peaks at 108.9 (C-1), 120.03 (C-2 and C-6), 138.7 (C-3 and C-5), 146.01 (C-4), were structurally indicative of the phenyl ring a sharp peak at 166.28 confirmed the presence of carbonyl group of the ethyl carboxylate group. Hence the structure of GAE isolated from the *Hippophae Rhamnoides* leaves was characterized with the help of the peaks and spectral data.

#### Fourier-transform infrared spectroscopic analysis

The IR spectra of GAE exhibited peaks at 3250 cm^−1^ due to a hydroxyl group, 1260 cm^−1^ due to C-O (phenolic group) on the aromatic ring. The peak at 2950 cm^−1^ due to the CH_2_ group was also found indicating the presence of the ethyl group. Another peak at 1700cm^−1^ was also present due to a carboxyl group. The NMR and IR data confirmed the presence and purity of GAE.

### Method development

To achieve the best chromatographic condition, a short reversed- phase Kinetex biphenyl 100 Å column (150 × 4.6 mm, 2.6 µm particle size) was selected. To optimize the best chromatographic conditions GAE was injected into the HPLC-system and several mobile phases were investigated by using a standard flow rate (0.5 mL/min). An adequate chromatographic condition was achieved on mobile phase composed of ACN (A) and water with 0.1% OPA (pH 3.3), (20:80 *v/v*) provide better resolution and sharp peak with satisfactory sensitivity.

### Selectivity

The endogenous interference was evaluated from the chromatograms derived from the blank samples, spiked samples, and samples after GAE administration. The chromatograms of GAE, plasma and tissue homogenates (liver, kidney, heart, spleen, and brain) spiked with IS and with different treatment group. Under the optimized chromatographic condition, the retention time of GAE was about 6.7 min, and no interference peak was detected at related times in blank plasma, tissues, and sample after GAE administration. No interfering peak was observed at the retention time of GAE and IS in all conditions.

### Linearity and the Limit of Quantitation (LOQ)

The linearity of the analytical method in the calibration range of 1–500 ng/mL demonstrated for GAE in plasma and tissue homogenates. The correlation coefficient of GAE is (r^2^) > 0.997 and the typical equation for the calibration curve was as follows, Y = 270.66x + 10872, where y represented the GAE concentration (ng/mL) and X represented the GAE peak area.

Under the experimental condition described, the LOQ of the GAE was 5.0 ng/mL in all mice matrices with acceptable precision (≤20%) and accuracy ± 20%. The LOD was established at 1.05 ng/mL in plasma and at 5 ng/mL in tissue respectively. These limits are sufficient for the pharmacokinetic and tissue distribution study of GAE.

### Accuracy and precision

The precision and accuracy were assessed by determining quality control sample (QC) sample (n = 5) at three levels of concentration and the results were presented in (Table 1). The intra-day and inter-day precision were 0.03–6.8 in plasma and 1.8–5.6% in the liver for GAE. These results suggest that the procedure described above for HPLC system were satisfactory and the method was accurate, reliable and reproducible.

**Table 1:** Accuracy, Precision and recovery of the method used for the determination of GAE in mice plasma and liver. GAE stability in plasma and liver was estimated at room temperature for 24 h Short term stability ^a^, Long term stability^b^ studied following 2 week of storage at −20 °C; Freeze thaw stability was evaluated at three consecutive freeze thaw cycles (−20 °C to room temperature).

Sample	Level (ng/mL)	Accuracy (RE%) n = 5	Precision (%RSD)		Recovery %	Stability %		
			Intraday	Interday		Short terma	Long termb	Freeze thawc
Plasma	50	−7.5	1.91	6.8	120.5 ± 7.2	79.35 ± 1.37	70.32 ± 1.37	78.95 ± 2.25
	250	1.7	0.79	2.8	91.26 ± 15.4	89.67 ± 1.58	87.55 ± 0.635	90.06 ± 0.93
	500	0.006	0.039	4.0	99.22 ± 2.72	99.25 ± 0.120	92.01 ± 0.179	93.42 ± 0.37
Liver	50	−5.6	4.72	5.6	66.9 ± 22.2	66.9 ± 22.18	62.6 ± 24.5	64.8 ± 4.9
	250	−4.06	2.77	3.0	86.8 ± 1.79	86.8 ± 1.7	79.26 ± 6.06	84.16 ± 2.5
	500	1.4	1.85	1.90	103.5 ± 0.005	99.9 ± 0.47	95.13 ± 7.6	97.83 ± 4.9

Pharmacokinetic parameter of GAE in mice plasma after oral administration of GAE (200 mg/kg body weight) (Table 2) and whole body exposure of ^60^Co-γ radiation at (5 Gy) + GAE (Table 3).

### Recovery and Stability

The recovery of GAE at three concentration was 79.3, 89.6 and 99.2% in plasma. The stability of GAE was found to be stable at room temperature for 24 h in plasma and tissue samples. GAE was found to be stable at −20 °C for one month. Moreover; GAE was found to stable after freeze-thaw cycle with a reduction of less than 8%. The analyte were also shown to be stable in the plasma at room temperature for atleast 8 h with a reduction of less than 12–15% (Table 1). Based on the result reliable, reproducible and robust method has been developed and validated.

### Pharmacokinetic and Biodistribution studies

The developed method was successfully employed to determine GAE concentration in mouse plasma and tissue samples after single oral administration of GAE at a single dose (200 mg/kg body weight) in GAE and Radiation + GAE (R + GAE) treated group. The non-compartmental models was found to be the best to fit the data. Pharmacokinetic parameter analysis is presented in (Table 2, Table 3). After oral administration of GAE at a dose of 200 mg/kg b.wt, the maximum concentration (C_max_) of GAE in GAE treated plasma was 4390.75 ± 285.20 and in R + GAE 3391.78 ± 78.13 ng/mL. The pharmacokinetic parameter area under the curve (AUC_0-t_) and (AUC_0-α_), T_max_, t_1/2_, and K_el_ in GAE-treated and R + GAE treated groups are listed in Table 3, indicating that the exposure of ionizing radiation reduces the maximum plasma GAE concentration of (C_max_) and increases the oral clearance (Cl/F) of GAE to 0.069 ± 0.01, in comparison to that of the GAE-treated group (0.017 ± 0.005).

**Table 2:** Pharmacokinetic parameter of GAE treated group in mice plasma.

Parameters	Value
C_max_ (ng/mL)	4390.85 ± 285.20
T_max_ (min)	15 ± 4.2
AUC_0-t_ (min.ng/mL)	175228.24 ± 5224.7
AUC_0-α_ (min.ng/mL)	175730.37 ± 5319.3
K_el_	0.026 ± 0.003
T_1/2_ (min)	26.54 ± 3.2
Clearance (mL/min)	0.017 ± 0.0005

**Table 3:** Pharmacokinetic parameter of R + GAE group in mice plasma.

Parameters	Value
C_max_ (ng/mL)	3391.78 ± 78.13
T_max_ (min)	15 ± 4.6
AUC_0-t_ (min.ng/min)	64379.3 ± 3812.5
AUC_0-α_ (min.ng/min)	64423.5 ± 7097.4
K_el_	0.025 ± 0.02
T_1/2_ (min)	22.38 ± 3.6
Clearance (mL/min)	0.07 ± 0.01

**Figure 2:**
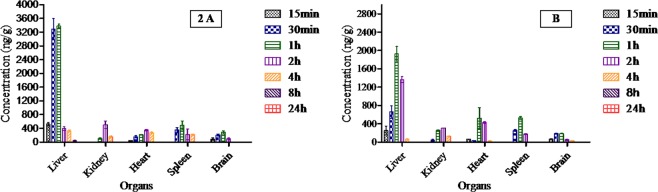
**(A)** Bio distribution of GAE in liver, kidney, heart, spleen and brain after 15 min, 30 min, 1 h, 2 h,4 h,8 h and 24 h following after oral administartion of GAE at a dose of 200 mg/kg b.wt. **(B)** Bio distribution of GAE after ^60^Co-γ radiation (5 Gy) + GAE (200 mg/kg b.wt) administration.

The Biodistribution in mice at 15 min, 30 min, 1 h, 2 h, 4 h, 8 h and 24 h after GAE administration are presented in (Fig. 2A,B). The data show that GAE is distributed widely in many tissues including liver, kidney, heart, spleen, and brain. Moreover; no GAE peak was identified in untreated control and irradiated animals. In the GAE treated group the concentration of GAE is highest in the liver 3383.1 ± 137.79 ng/g at 1 h, 529.13 ± 102.8 ng/g in the kidney at 2 h and 283.1 ± 20.60 ng/g in the brain at 1 h after GAE administration. In the R + GAE treated group the concentration of GAE was reduced at 1928.5 ± 39.63 ng/g, when compared to the GAE-treated group animals. The kidney concentration was also reduced 307.77 ± 7.80 ng/g in the R + GAE treated group. In R + GAE group the GAE concentration increased from 216 to 506 ± 39.4 ng/g in the heart and from 484 to 522 ± 73.20 ng/g in the spleen at 1 h when compared to GAE treated animals. In both the GAE treated and the R + GAE treated groups, the peak GAE concentration in liver was two fold higher than in other tissues. After 2 h of GAE administration its concentration decreased GAE was found in heart, spleen, and kidney up to 4 h, whereas in brain it found in low concentration up to 2 h. Notably, GAE was detected in the brain at appreciable concentration in both GAE and R + GAE-treated groups, and suggesting that GAE could cross the blood-brain barrier.

### Effect of GAE on liver and kidney function markers SGOT, SGPT, Urea and creatinine

**Figure 3:**
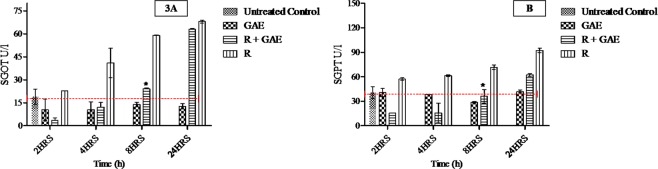
**(A)** Modifying action of GAE on SGOT level in liver of whole body ^60^Co-gamma irradiated mice. *P < 0.05, when compared with irradiated animals. **(B)** Modifying action of GAE on SGPT level in liver of whole body ^60^Co-gamma irradiated mice. *P < 0.05, when compared with irradiated animals.

**Figure 4:**
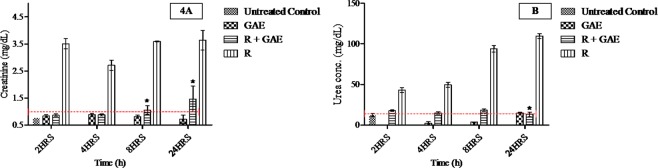
**(A)** Modifying action of GAE on creatinine in kidney of whole body ^60^Co-gamma irradiated mice. *P < 0.05, when compared with irradiated animals. **(B)** Modifying action of GAE on urea level after whole body ^60^Co-gamma irradiated mice. *P < 0.05, when compared with irradiated animals.

Whole body exposure of ^60^Co-γ radiation at a 5 Gy dose caused a significant change in markers of a liver injury such as SGOT, SGPT (Fig. 3A,B) as well as changes in kidney markers such as urea and creatinine (Fig. 4A,B). Oral administration of GAE at a dose rate 200 mg/kg b.wt post 15 min of irradiation showed a significant reduction (*P < 0.05) of these markers in the serum for R + GAE and GAE-treated groups, when compared to animals irradiated but not receiving GAE.

### Measurement and Quantification of Nuclear factor kB (NF-kB) in liver and kidney by using indirect ELISA and immunohistochemistry

**Figure 5:**
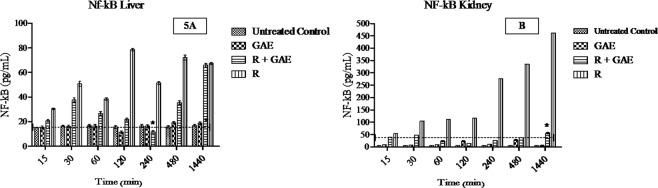
NF-kB level in liver (**A**) and kidney (**B**) of mice treated with GAE, R, R + GAE. The data was collected at different time point, dashed line represent value obtained from untreated animals. Each value represent mean + standard deviation (SD). *Represents significant difference with respect to untreated control at p < 0.05.

**Figure 6:**
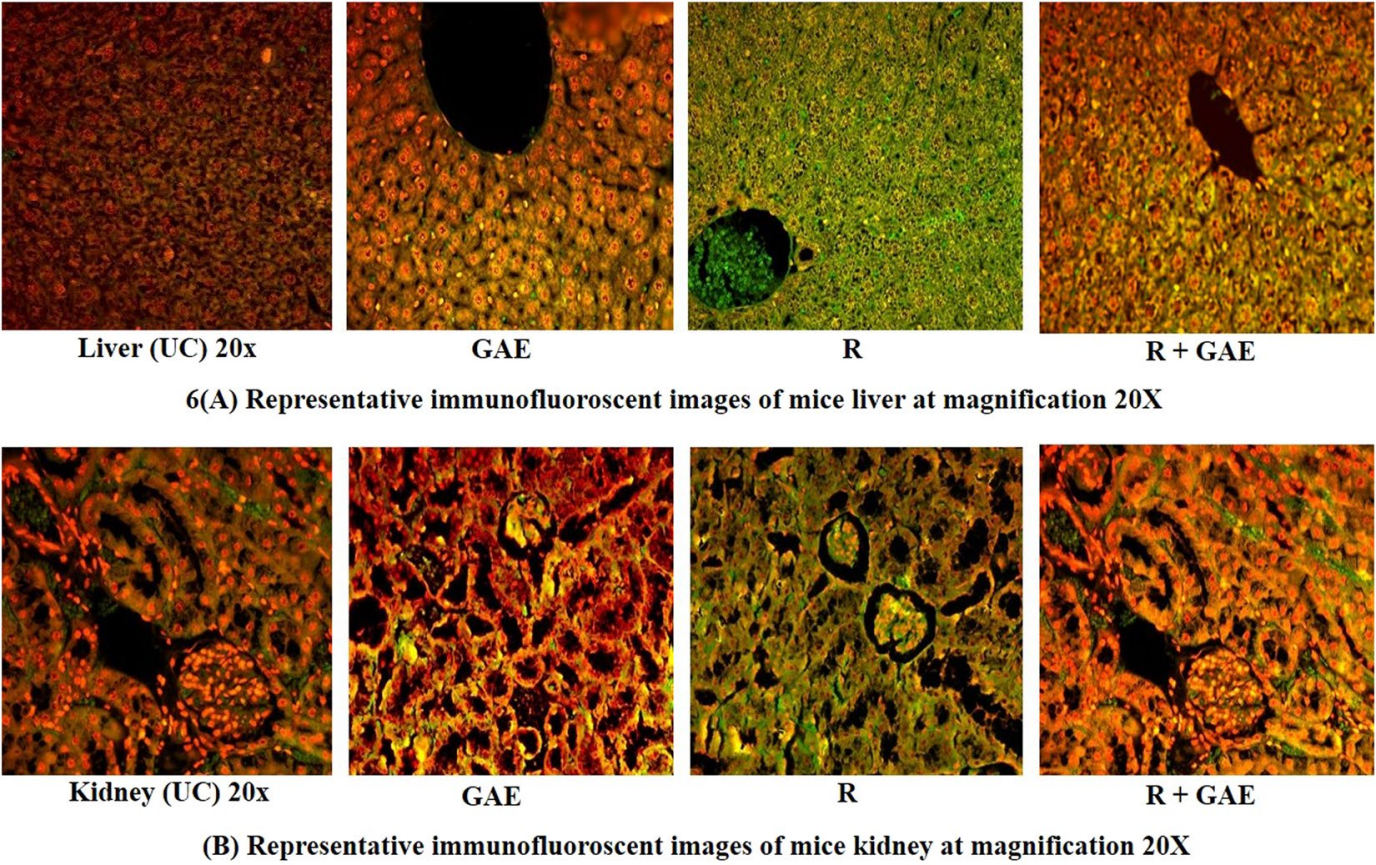
Representative Immunofluoroscent images of liver and kidney of mice treated with GAE, Radiation (R), R + GAE. NF-kB overexpressed in irradiated group present with positive green color. **(A)** Immunofluoroscent images of liver at 20X untreated control (UC), GAE, R and R + GAE. **(B)** Immunofuoroscent images of kidney at 20X UC, GAE, R and R + GAE.

NF-kB is a key mediator pro-inflammatory cytokines play a significant role in inflammation induce after radiation exposure. The time-dependent study showed major expression of NF-kB in liver and kidney after irradiation. The GAE treated animal showed significant minimal expression (p < 0.05) in comparison to irradiated animals. A significant increase in the NF-kB level showed a positive correlation with inflammation. NF-kB levels were quantified by using indirect *ELISA* (Fig. 5A,B). NF-kB expression was also confirmed by means of immunofluorescence staining. NF-kB positive fluorescence was strongly expressed in the central vein, hepatocytes and sinusoidal gaps of liver sections in irradiated mice. Moreover, the NF-kB positive fluorescence expression was not strong in R + GAE group. Similarly, the NF-kB positive fluorescence expression was expressed in tubules, glomeruli, Bowman capsules and proximal and distal tubules in the kidney section of irradiated mice. The expression was higher in the irradiated group in comparison to the R + GAE group (Fig. 6A,B).

## Discussion

Although GAE has many pharmacological properties due to its polyphenol in nature its radiomodifying action has not been reported to date. GAE isolated from *Hippophae rhamnoides* leaves and further characterized with NMR spectra indicate the presence of the CH_3_ group confirmed the purity of GAE. To the best of our knowledge, this is the first study reporting the pharmacokinetic and biodistribution of GAE and the changes in biodistribution and pharmacokinetics of GAE induced after whole-body exposure of ^60^Co-γ radiation at a dose (5 Gy). Previously several sulfhydryl and synthetic compounds were screened and tested *in-vitro* and preclinical models for developing potent radiation countermeasure but few of them has been showed better result in animal models but found toxic in clinical application^16,17^. Our study has demonstrated the anti-inflammatory and radiomodifying effect of GAE after few minutes to hours of ionizing radiation exposure. NF-kB is one of the key regulator responsible for the activation and transcription of proinflammatory cytokines which induce inflammation. NF-kB also play an important role and prompt the inflammatory disorders *via* mediating pro-inflammatory cytokines, chemokines, adhesion molecule etc^18,19^. Administration of GAE mitigates NF-kB expression in liver and kidney found in irradiated mice, as assessed by indirect ELISA. Moreover, GAE administered 15 min after whole body ^60^Co-γ radiation exposure (5 Gy) significantly inhibits NF-kB expression at cellular level upto 8 h. Immunofluorescence staining of liver and kidney sections were performed to check expression of NF-kB in the R + GAE group, which was not positive as compared to the irradiated group. A method for the quantitative estimation in plasma and tissue of GAE in mice was successfully developed by using a high-performance liquid chromatography (HPLC). This method has the advantage of being sensitive, specific and accurate, and it can be used for pharmacokinetic studies in rodent models. Prior to injecting the sample into the HPLC, the maximum wavelength for GAE was determined spectrophotometrically and the absorbance was recorded 270–285 nm. For standardizing the chromatographic conditions, different influencing factors such as different ratios and composition of the mobile phases, as well as different chromatographic columns were evaluated. Previous evidence suggested that the use of ACN and water with 0.1% orthophosphoric acid (OPA), pH 3.3 (20:80 v/v) provide better resolution and sharp peak of GAE and IS with satisfactory sensitivity. A short reversed-phase Kinetex biphenyl C_18_ Phenomenex 100 Å column (150 × 4.6 mm, 2.6 µm particle size) showed better resolution with retention time 6.7 min. One unknown metabolite was observed in all the samples at retention time 3.7 min. Prior to injecting the plasma and tissue homogenates, bio-samples pretreatment was determined to remove the exogenous and endogenous interferences in HPLC. Plasma and tissue homogenate protein precipitation was accomplished by the addition of organic solvent ACN. The use of plasma and ACN at 1:3, tissue homogenate and ACN at 1:2 give a better result. The sample was centrifuged at 4 °C, which precipitated the protein. In addition, the plasma and tissue sample was filtered and reconstituted with mobile phase and the supernatant was injected into the HPLC system with a standard flow rate of 0.5 mL/min.

Successful method validation allowed determination of the pharmacokinetic of GAE after an oral administration. Our research study indicates that administration of GAE in C 57 BL/6 mice prior and post radiation showed the major difference in C_max_ as well as in biodistribution. The plasma AUC is fit to a non-compartmental model, and presents these differences. The absolute biodistribution of GAE after a 5 Gy dose of radiation significantly reduced the GAE concentration in the liver and kidney when compared to the corresponding volume in the GAE-treated group’s animal group. After oral administration of GAE its concentration decreased with time due to phase 2 metabolism, which also relates to its polyphenol nature, as well as its rapid hydroxylation, which occur during the stabilizing of free radicals induced by the radiation exposure. The Hydroxyl group on GAE donate their protons to a reactive oxygen species to quench and stabilize the radicals in the body after radiation exposure. The hydroxylation at a particular position on GAE also enhances its plasma protein binding affinity. Moreover, the esterification or presence of ethyl ester with Gallic acid increases its binding affinity to plasma proteins^20,21^. C_max_, AUC_0-t_ of plasma in R + GAE mice was smaller as compared to the GAE treated group. The higher C_max_ of GAE in the GAE-treated group showed better absorption whereas reduction of GAE C_max_ in R + GAE group showed the decrease of absorption along with an increase in Cl/F. A concomitant increase in GAE clearance was observed in R + GAE groups, due to changes in pathophysiological states and damage in endothelial cells and gastrointestinal damage after radiation exposure. Moreover; the GAE was found in brain of mice in both groups which may indicate that it the crosses the blood brain barrier. Similarly the presence of GAE in *Terminalia chebula* extract showed neuroprotective properties^15^. In the R + GAE group GAE mitigate the damage induced by radiation exposure at the cellular and tissue level. GAE significantly reduces the NF-kB level in liver and kidney when compared to irradiated mice not receiving GAE. The result shows that GAE is a promising potential inhibitor of the inflammatory mediator’s response, which is associated with chronic oxidative stress induced by radiation exposure. Moreover, GAE also showed the radiomodifying action by attenuating the increase in SGOT, SGPT, urea and creatinine level in R + GAE group that occur post radiation exposure. The SGOT and SGPT level at 24 h in R + GAE was 63.11 ± 6.16, 62.15 ± 8.63 at 24 h, 24.10 ± 2.4, 36.01 ± 6.1 at 8 h and in radiation (R) group 68.02 ± 8.6, 92.36 ± 3.70 at 24 h, 68.02 ± 1.2, 71.70 ± 6.1 U/L at 8 h. Moreover; the urea level in R + GAE was 18.16 ± 0.003, and in radiation group was 94.01 ± 0.01 at 8 h whereas creatinine level in R + GAE was 1.05 ± 0.04 and in radiation group 3.5 ± 0.01 mg/dL. Values were mostly similar in comparison to Radiation group in SGOT and SGPT at 24 h. The result showed that GAE showed radiomodifying action upto 8 h and beyond them its radiomodifying efficacy slowly reduced due to changes in their different metabolites or changes in structure post irradiation. The result showed that post irradiation GAE modify the biomarker of liver and kidney function of mice upto 8 h, moreover at 24 h the GAE radiomodifying efficacy was reduced. Our experimental study suggests the possibility of making changes to the GAE formulation to further improve GAE absorption and enhance pharmacokinetic characteristic achieve better efficacy post radiation exposure in medical applications or in of radiation threat situations.

## Material and Methods

### Chemical and reagents

Propyl gallate (IS) Sigma, India, Methanol (HPLC grade) were purchased from Merck (Mumbai India), Ultra-pure water (HPLC grade 18 MV) purchased from Thermo scientific, Acetonitrile (HPLC grade), orthophosphoric acid (OPA), Hydrochloric acid (HCl) were purchased from Merck (Mumbai, India). Serum glutamate oxaloacetate transaminase (SGOT, S.L), serum glutamate pyruvate transaminase (SGPT, S.L), Urea U.V (S.L) and creatinine kits were purchased from agape Kerala, India. All other chemical and solvent used of analytical grade. NF-kB enzyme-linked immunosorbent assay (ELISA) kit was purchased from BD Bioscience (USA), NF-kB primary antibody for Immunohistochemistry PAB699Mu01, Mus musculus (specific) was purchased from Cloud-Clone Corporation, USA. Formaldehyde, hematoxylin and eosin stains and other analytical grade chemical and reagents for HPLC and phytochemical analysis were purchased from Sigma Pvt., Ltd., India.

### Isolation, Extraction, and Characterization

The aerial part of *Hippophae rhamnoides* leaves were powdered finely and extracted with 95% ethyl alcohol, filtered and dried under reduced pressure in rotavapor. The dried extract was dissolved and filtered, the filtrate was treated with an aqueous solution of sodium carbonate. The residue of the filtrate was neutralized with an aqueous solution of HCl n/10 = (1n). After keeping for some time the precipitate obtained was filtered and washed with cold water to remove the traces of HCl. The solid residue obtained was recrystallized from the minimum quantity of boiling water, on cooling the crystal will appear and crystal was collected, dried and their melting point was recorded.

The isolated compound GAE was further characterized by using ^1^H NMR (400 MHZ) and ^13^C were obtained on Bruker DRX 400 spectrophotometer in Dimethyl sulfoxide (DMSO) using TMS as an internal standard reference, chemical shift in δ (ppm) and coupling constants (J values) is mention in Hertz. The FTIR spectra of GAE was recorded using a Nicolet 8700 spectrometer equipped with a DTGS-TEC detector and the data were analyzed on OMNIC software. The scan was collected at a resolution of 4 cm^−1^ and a data spacing of 1.928 cm^−1^ in order to achieve sufficient signal to noise ratio.

### Experimental animals, GAE administration, irradiation, and sampling

Black C57BL/6 male mice weighing 28–30 g were maintained at the Institutional Animal Experimental Facility. Animals were fed according to standard chow diet having free access to food and water *ad libitum*. The environmental condition was maintained at room temperature (22 ± 2 °C), relative humidity (45–60%) and 12 h dark/light cycle controlled facility. In this study, mice were randomly assigned to four different groups (n = 21) *i.e* untreated control (UC), GAE-treated (GAE), Radiation + GAE (R + GAE), Radiation group (R). Mice fasted overnight with free access to water before being dosed. Isolated GAE from leaves was dissolved with distilled water and ethanol in (7:3 v/v) and administered orally at a dose of 200 mg/kg body weight to GAE-treated and R + GAE treated groups only. The experiment was carried out in accordance to regulation and guideline of the, Committee for the purpose of control and supervision of Experiments on animals (CPCSEA), and study was approved by Institutional Animal Experimental Committee (IAEC), Institute of Nuclear Medicine & Allied Science, Delhi, India.

Group I untreated control (UC), Group II GAE-treated (GAE), Group III Radiation + GAE (R + GAE) and Group IV Radiation (R) exposed. The GAE was administered orally 15 min post irradiation. The whole-body radiation (5 Gy) was carried out using Bhabahtron II, Telecobalt machine, Panacea medical technology, Bangalore. The mice exposed at a dose rate of 0.961 Gy/min at field 35 × 35 cm^2^ and the distance maintained from the source was 80 cm.

Blood and tissues samples of mice were collected at 15 min, 30 min, 1 h, 2 h, 4 h, 8 h and 24 h after GAE administration (3mice per time). Blood was obtained from retro orbital plexus of mice and collected in heparinized eppendorf tubes, followed by centrifugation at 2500 g for 15 min. The resulting plasma layer was separated and stored in tubes at −20 °C for further analysis. Tissue samples (brain, heart, liver, kidney, and spleen) were weighed and put into the normal saline solution to remove the blood by blotting on filter paper and were minced and homogenized with physiological saline solution (1:2 w/v) thoroughly in an ice bath. The homogenates were stored at −80 °C until the analysis.

### Measurement of Serum glutamic oxaloacetic transaminase (SGOT), Serum glutamic pyruvic transaminase (SGPT), Urea and creatinine level

SGOT and SGPT are a biomarker of liver function test used to assess liver function and its damage; whereas, urea and creatinine were used to investigate kidney function and its damage. These biomarkers were analyzed *in-vitro* by the use of commercially available kits of agape, Kerala, India. The assay were performed according to standard procedure mention in the kit protocol. *In-vitro* SGOT quantitative determination were based on the reaction of L-aspartate with alpha-ketoglutarate. According to the manufacturer protocol reagent were mix and incubated for 1 minute at 37 °C. The changes in absorbance were recorded per minute during 3 minutes. *In-vitro* kinetic determination of SGPT were based on the reaction of L-alanine with alpha-ketoglutarate. The reagent were mixed and incubated for 1 minute at 37 °C. the changes in absorbance was recorded per minute during 3 minutes at 340 nm. Urea is *in-vitro* quantitative determine by the method of Talke *et al*., 1965. Creatinine were *in-vitro* determined by kits and it can be measured by quionone pigment photometrically. Reagent were mixed and incubated for 5 minute at 37 °C. the sample absorbance and the standard against the reagent blank were measured at 546 nm.

### Quantification of NF-kB activity by using Indirect ELISA

Enzyme-linked immunosorbent assay was used to estimate the nuclear factor k beta (NF-kB) in the liver and kidney of mice. Measurement of NF-kB activity was done in accordance to the manufacturer’s protocol purchased from BD-biosciences. 100 µl of primary antibody NF-kB coated on 96 well micro plates and incubated overnight at 4 °C. Plates was washed three time with wash buffer and were blocked with 200 µl blocking buffer per well and incubated for2h at room temperature. In each well tissue sample were added and incubated for 1 h at room temperature. After development of colour 50 µl of stop solution was added to the wells and absorbance was recorded at 490 nm. A standard curve was prepared from the data produced from the serial dilution of the pure NF-kB proteins with concentration on the x-axis (log scale) vs absorbance on the Y-axis (linear).

### Immunohistochemistry of NF-kB expression

Immunofluorescent staining of nuclear factor –kB (NF-kB) was performed by using formalin fixed paraffin embedded tissue by using standard procedure and protocol. The liver section 5 µm were immunohistochemically stained. Primary polyclonal antibody mouse to Nuclear factor (NF)-kB (1:200) was used. After washing, the slides were counterstained with ethidium bromide (EtBr) and incubated for 1 h at room temperature. NF-kB activity was visualized under a fluorescent microscope Axioscope A1 make Carl Zeiss. The images was visualized and captured by the use of Axiovision software.

### GAE extraction and sample preparations

Aliquot of plasma sample (100 µL) dispensed in standing vial deproteinized with ACN (200 µL) containing the IS (100 ng/mL) and centrifuged at 2500 g for 15 min at 4 °C. The supernatant was collected transferred to a clean glass tube, reconstituted with 200 µL mobile phase vortexed mixed for 2 min and centrifuged at 2500 g for 5 min. Following this, a 20 µL aliquot of supernatant was injected into the HPLC system for analysis.

For the extraction from tissue, each aliquot (300 µL) of homogenate (liver, kidney, brain, heart & spleen) was spiked with 20 µL of the IS working solution (100 ng/mL) during analysis; homogenate was dispensed in a standing vial containing 400 µL ACN to precipitate proteins. The biological samples were swirled for 3 min and were centrifuged at 12000 rpm for 10 min at 4 °C. The obtained supernatant was tubed and dried under a stream of nitrogen at 40 °C, the sample was reconstituted with mobile phase, then and 20 µL was injected into the HPLC system.

### Method Validation

GAE was separated on the high-performance liquid chromatography (HPLC), SPD-10A consisting of binary HPLC pump, ultraviolet detector, and a Rheodyne manual injector equipped with a 20 µl loop (Shimadzu, Japan). Separation was achieved on Kinetex Biphenyl RP 100 Å column (2.6 µm, 150 × 4.6 mm) (Phenomenex, Torrance) installed with phenyl cartridges (Phenomenex, Torrance). The system was controlled using class VP software (Shimadzu, Japan). The chromatographic separation of GAE was in approximately within 10 min, at room temperature by using the isocratic mobile phase mixture of ACN and 1%OPA (30:70, v/v) with pH maintained at 3.3. 20 µL of the samples were injected at a flow rate of 0.5 mL/min and the wavelength 270 nm was used for detection.

Standard stock solutions of GAE at 1 mg/mL were prepared and dissolved in appropriate amounts of diluent stock, water, and methanol (8:2 *v/v*). GAE was adequately diluted with diluent stock to give an intermediate solution at 100 µg/mL for each analyte. GAE calibration standards were at concentration of 5, 10, 20, 30, 40, 50, 100, 250, 500 ng/mL. Internal standard propyl gallate (PG) were prepared to spiked aliquots of blank mouse plasma (200 µL). Seven combined spiking solution at a final concentration of 5, 10, 20, 40, 50, 100, 250, 500 and 1000 ng/mL were prepared and used to spike aliquots of blank tissue homogenates of Liver, kidney, heart, brain, and spleen (400 µL). The entire sample was stored at 4 °C. Quality control samples were prepared independently in the same biological matrices (plasma and tissue homogenate of liver) stored at −20 °C until use with each analytical run. Intra-day and inter-day precision were assessed by using six replicate QC samples at three levels of GAE included in each run to determine the accuracy and precision. The acceptance criterion for intra-day and inter-day precision did not exceeded 15%. The inter-day accuracy and precision were assessed by analyzing three batches on different days. The recoveries of GAE from plasma and tissue (liver) homogenate samples were done at three QC levels. The recovery of the analyte was calculated by comparing the analyte peaks of extracted QC samples against the equivalent aqueous solution.

The QC samples were kept at room temperature for 6 h and a freeze-thaw cycle (−80 °C) was repeated for three times. The QC sample was stored for 30days at −80 °C respectively. For all storage conditions, replication of three concentrations was analyzed after the operation and the experimental result was obtained through a chromatographic area and compared with the nominal values. On the basis of previous studies reported on pharmacokinetic and biodistribution^22,23^, we standardized and validated our study to further investigate the radiomodifying action of GAE.

### Statistical analysis

The pharmacokinetic parameters of GAE were obtained with the help of a computer designed program PK solver add-in macros programme in Microsoft Excel and other marker assay and biodistribution were analyzed using one-way ANOVA with Bonferroni post-T-test comparison using GraphPad Prism software (5.01).
